# Supplementary material for: Impact of antigen test target failure and testing strategies on the transmission of SARS-CoV-2 variants
Source: Nat Commun. 2022 Oct 5;13:5870. doi: 10.1038/s41467-022-33460-0 (PMC9533294; doi:10.1038/s41467-022-33460-0)
Supplement: Supplementary file 1 — Supplementary Information [file 41467_2022_33460_MOESM1_ESM.pdf]

Supplementary Information
Impact of antigen test target failure and testing strategies on the transmission of SARS-CoV-2 variants

Claudia Del Vecchio[1^], Bethan Cracknell Daniels[2^], Giuseppina Brancaccio[1], Alessandra Rosalba Brazzale[3], Enrico Lavezzo[1], Constanze Ciavarella[2], Francesco Onelia[4], Elisa Franchin[4], Laura Manuto[1], Federico Bianca[1], Vito Cianci[5], Anna Maria Cattelan[6], Ilaria Dorigatti[2,§,*], Stefano Toppo[1,7,§,*], Andrea Crisanti[1,4,8,§,*]

[1]Department of Molecular Medicine, University of Padua, Via Gabelli, 63, Padua 35121 Italy
[2]MRC Centre for Global Infectious Disease Analysis and Jameel Institute, School of Public Health, Imperial College London, London, UK
[3]Department of Statistical Sciences, University of Padua, Via C. Battisti 241, Padua 35121, Italy
[4]Microbiology and Virology Diagnostic Unit, Padua University Hospital, Via Giustiniani 2, Padua 35128 Italy
[5]ER Unit, Emergency-Urgency Department, Padua University Hospital, Via Giustiniani 2, Padua 35128 Italy
[6]Infectious and Tropical Diseases Unit, Padua University Hospital, Via Giustiniani 2, Padua 35128 Italy
[7]CRIBI Biotech Center, University of Padua, V.le G. Colombo,3, Padua 35131 Italy
[8]Department of Life Science Imperial College London, South Kensington Campus, Imperial College Road, SW7 AZ London, United Kingdom

^ these authors contributed equally
§ these authors jointly supervised this work

* Corresponding authors:
Andrea Crisanti: a.drcrisanti@imperial.ac.uk
Stefano Toppo: stefano.toppo@unipd.it
Ilaria Dorigatti: i.dorigatti@imperial.ac.uk

Contents

Evaluation of sample selection bias 3
Stratification of test performance 4
Supplementary Figures 5
Supplementary Figure 1 | Distribution of tests by age 5
Supplementary Figure 2 | Distribution of test type by age 6
Supplementary Figure 3 | Sensitivity estimates of antigen test according to Ct values 7
Supplementary Figure 4 | Distribution of Ct values 8
Supplementary Figure 5 | Monthly prevalence of the detected variants obtained from the GISAID database 9
Supplementary Figure 6 | Transmission dynamics of SARS-CoV-2 variants (A220V, M234I-A376T, Alpha and other) in Veneto and the rest of Italy under different testing scenarios. a) Veneto and b) the rest of Italy estimated daily reported incidences per 100,000 population under the baseline testing scenario (.......... 10
Supplementary Figure 7 | Flow diagram of the transmission model used to reproduce the dynamics of the discordant variant M234I-A376T, the concordant variant A220V, the alpha variant and other variants, in Veneto and Italy. 11
Supplementary Figure 8| Distribution of Ct values according to days from symptom onset 12
Supplementary Figure 9| Distribution of Ct values above and below 33 according to time intervals 13
Supplementary Tables 14
Supplementary Table 1 | Estimates of antigen test sensitivity according to Ct values. 14
Supplementary Table 2 | Distribution of Ct values of samples. 15
Supplementary Table 3| Antigen sensitivity against variants with and without the amino-acid substitutions M234I and A376T, mapping to regions of the SARS-CoV-2 N protein containing epitopes that function as the target of capture antibodies in antigen tests. 16
Supplementary Table 4 | Antigen sensitivity against variants with and without any mutation mapping to regions of the SARS-CoV-2 N protein containing epitopes that function as the target of capture antibodies in antigen tests. 16
Supplementary Table 5 | Description of the concordant and discordant variants. 16
Supplementary Table 6| Posterior mean and 95% CrI parameter estimates for model variants assuming different testing scenarios. Estimates are obtained from the 2.5% and 97.5% percentiles of the posterior distributions. 17
Supplementary Table 7| Posterior mean and 95% CrI parameter estimates for model sensitivity analyses. ϕ_{Ag} = Antigen test sensitivity; $1/\eta$ = latency period; $1/\sigma$ = pre-symptomatic infectious period; $1/\gamma$ = infectious period; ζ = vaccine efficacy against infection; α = reduction in transmission of symptomatic infectious individuals due to reduced exposure. Estimates are obtained from the 2.5% and 97.5% percentiles of the posterior distributions. 18

Supplementary Table 8| Antigen test sensitivity according to days intervals from symptom onset. Test sensitivity at increasing time interval from symptom onset with 95% confidence intervals. P-value = 0.22 for equality of proportions. 20
References 21

Evaluation of sample selection bias

Of the 3,290 patients examined from the 15th of September to the 16th of October in the A&E and the Infectious Diseases wards, 1,441 (44%) were tested twice with both molecular and antigen tests, while 1849 patients (56%) were only tested once, with either molecular or antigen test. Individuals from 0-19 years old were predominantly tested with only one test (Figure S1) and mainly molecular (Figure S2), in line with the current school regulations which require a negative molecular test outcome for school re-admission. These individuals were, consequently, omitted from the analysis to avoid sampling biases. This reduced the analysis on 1,387 subjects who underwent both tests and 1,254 subjects who underwent only one test.

The 1,387 patients of our main study were compared to the 1,254 patients who, in the same observation period, were only given either a molecular or an antigen test. We did not find any clinically relevant difference with respect to sex (47% vs 53% females, P-value = 0.004), hospital ward of admission (80% vs 76% emergencies, P-value = 0.02) and, for the patients who tested positive, severity of the disease (P-value=0.77).

The majority of patients in age groups 20-49 underwent only one test; from age 50 onwards this trend reverses (Supplementary Figure 1). antigen is the preferred test type for patients who were only tested once except for age group 30-39 (Supplementary Figure 2). Further stratification by pneumonia did not show any significant difference (P-value = 0.37) although pneumonia was present in 48% of the antigen testing positives vs 17% of the molecular testing positives.

The monthly incidence of true positives, as benchmarked by a positive molecular test outcome, is 4.4% for those who received both molecular and antigen tests. The incidence lowers to 1.2% for patients who were only tested with molecular and 2.6% for those who were tested with antigen only. For patients with only the antigen test, the monthly incidence was estimated to be 3.8% using the overall specificity of 99.9% and sensitivity of 68.9% for the antigen test[1].

Stratification of test performance

We found no statistically significant association between the outcome of testing (i.e., discordance or concordance between antigen and molecular testing) with age, sex, and the presence of severe disease (pneumonia) at the time of testing. Moreover, there was no difference in the distribution of the Ct values for the S and ORF1 genes when comparing samples stratified by age, sex and disease severity The time interval between the date of symptom onset (known for all 51 symptomatic patients) and the date of testing ranges from 0 to 20 days, with a median of 2 and a mean of 4 days. The sensitivity of the antigen assay decreased from 75% (95% CI, 55-89%) to 60% (95% CI, 17-93%) to 50% (95% CI, 29 -71%) when the test was performed within 3 days, on days 4 or 5 and after 5 days after the development of symptoms (Supplementary Table 8). However, this trend is not statistically significant, possibly due the small sample size of each group (28, 5 and 18 patients). We found evidence that the Ct values of both genes increases in time from symptom onset to day of testing (Supplementary Figure 8 and Supplementary Figure 9, Pearson's correlation coefficient of the S gene: 0.34 with p-value = 0.016; Pearson's correlation coefficient of the ORF1 gene: 0.48 with p-value < 0.001). The sensitivity of the 10 asymptomatic patients is 90% (95% CI, 54-99%) with a Ct value for both S gene and ORF 1 gene below 33. Notably, these patients performed the test shortly after the diagnosis of infected relatives as part of the implemented surveillance measures.

Supplementary Figures

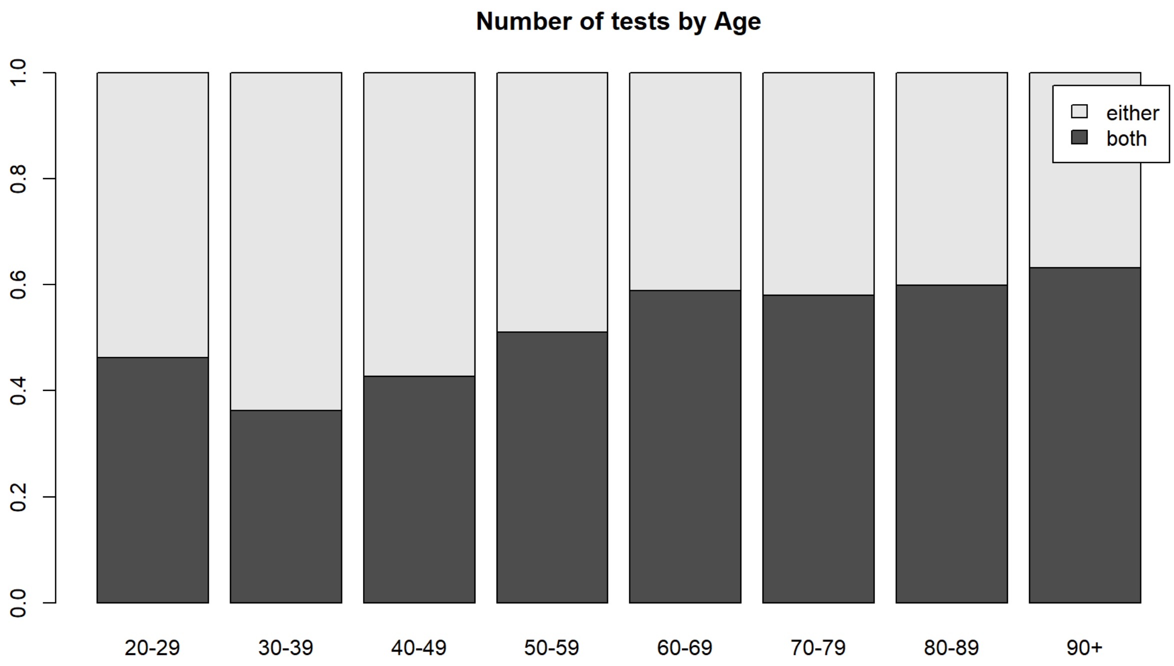

Supplementary Figure 1 | Distribution of tests by age: Percent stacked bar plot of the number of tests that were delivered in each age group. Individuals who performed only one test "either" molecular or antigen is reported in light grey. Subjects who performed both tests (molecular and antigen) are reported in dark grey.

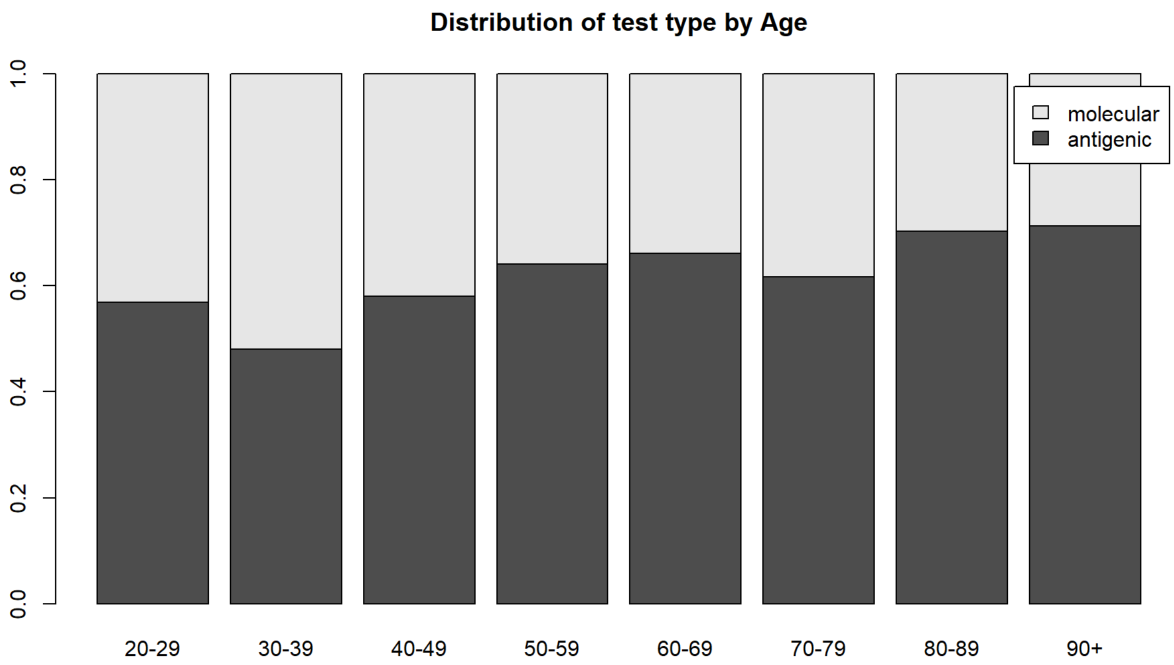

Supplementary Figure 2 | Distribution of test type by age: Percent stacked bar plot of the type of tests that were delivered in each age group. Individuals who performed only molecular are reported in light grey. Subjects who performed only antigen are reported in dark grey.

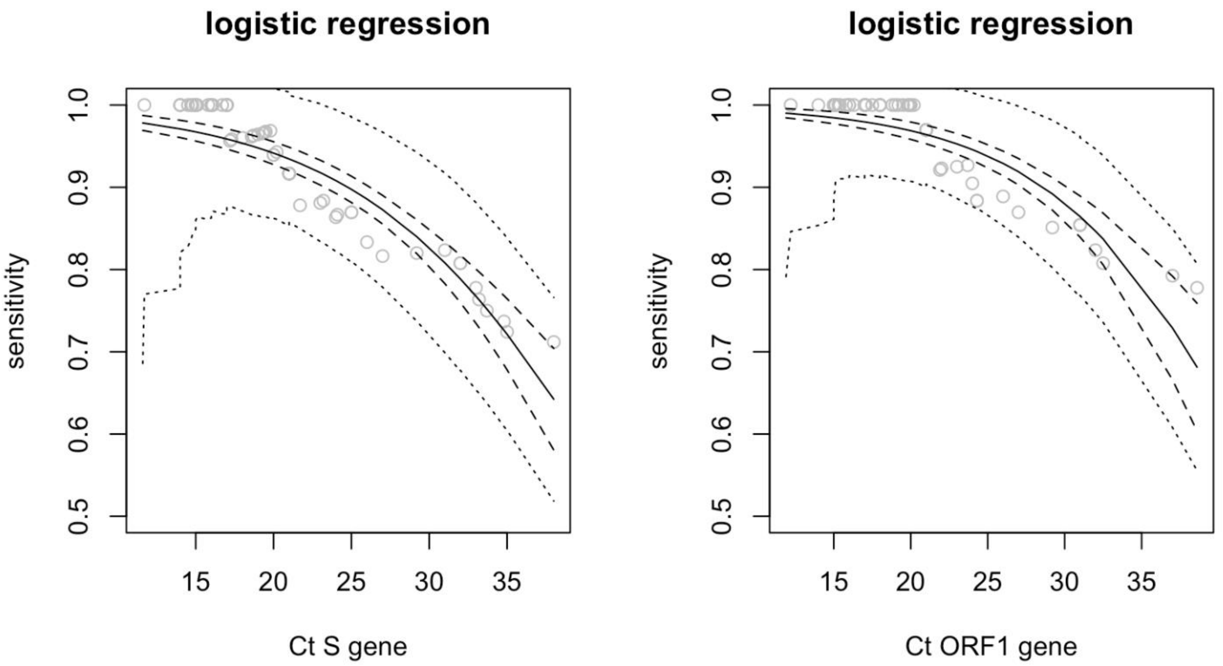

Supplementary Figure 3 | Sensitivity estimates of antigen test according to Ct values: Sensitivity estimates (solid line) of antigen test for increasing Ct values of the S gene (left panel) and Ct ORF1 (right panel) gene as obtained from a logistic regression model. The dashed lines give the 95% confidence band for the fitted regression line. The dotted lines represent the 95% band for the fitted sensitivities.

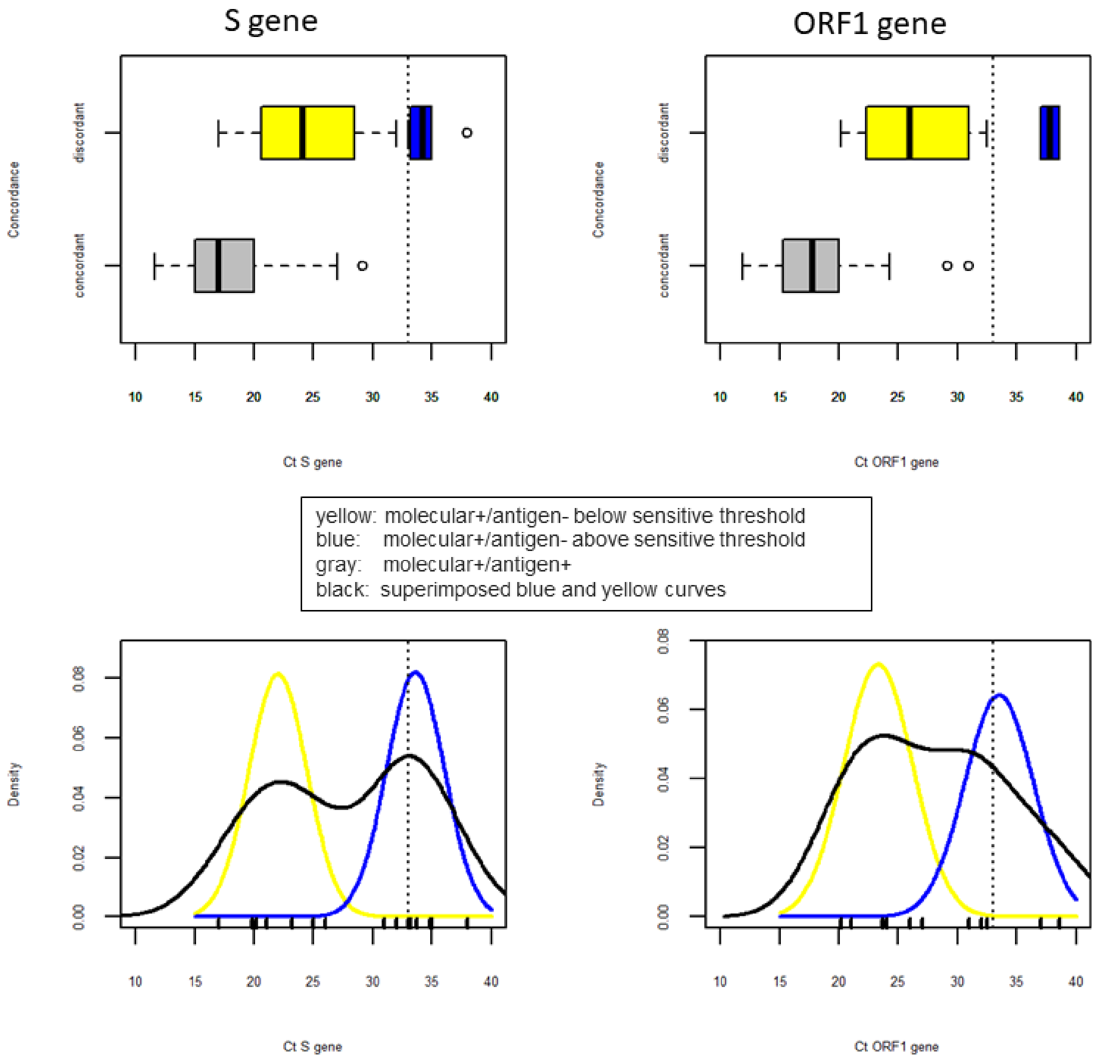

Supplementary Figure 4 | Distribution of Ct values: Distribution of Ct values for concordant molecular +/ antigen + and discordant molecular + / antigen - test results. Top row: Boxplots of Ct values for the S (left) and ORF1 (right) gene. Dotted line at Ct = 33 shows the critical value of sensitivity (95%) declared for the antigen test above which there is an expected drop and an unreliable result. molecular +/ antigen + (grey box), molecular +/ antigen - below sensitive threshold (yellow box) molecular +/ antigen - above sensitive threshold (blue box). Box plots show the centre line as the median, box limits as the lower and upper quartiles, whiskers as 1.5 times the interquartile range or, if no points exceed this distance, as minimum and maximum values. Where whiskers are 1.5 times the interquartile range, points show data values exceeding this range. Bottom row: Nonparametric density estimation for Ct values of the S (left) and ORF1(right) gene, with superimposed the two densities fitted using a two-component Gaussian mixture model. molecular +/ antigen - below sensitive threshold (yellow line) molecular +/ antigen - above sensitive threshold (blue line). n=61 biologically independent test samples.

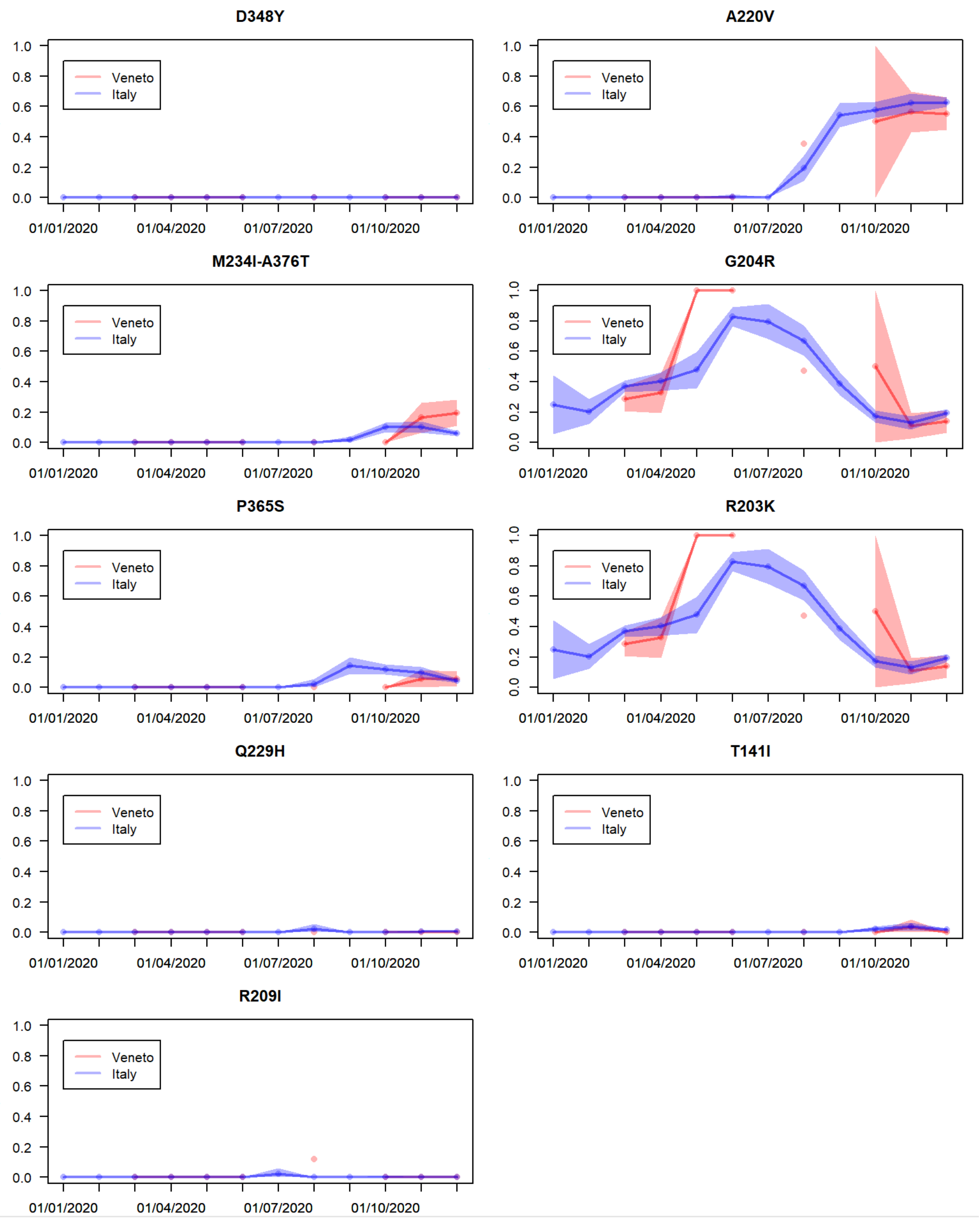

Supplementary Figure 5 | Monthly prevalence of the detected variants obtained from the GISAID database. Monthly prevalence (percentage over total sequences) of N variants generating discordant molecular + / antigen - (left panels) and concordant molecular + / antigen + (right panels) result in molecular and antigen swab. Solid line is the mean and shaded areas are the 95% confidence bands for Veneto and the rest of Italy. No sequence data are available for Veneto region in the months of January, February, July, and September. To note that A220V, G204R and R203K mutations appear in concordant and discordant samples.

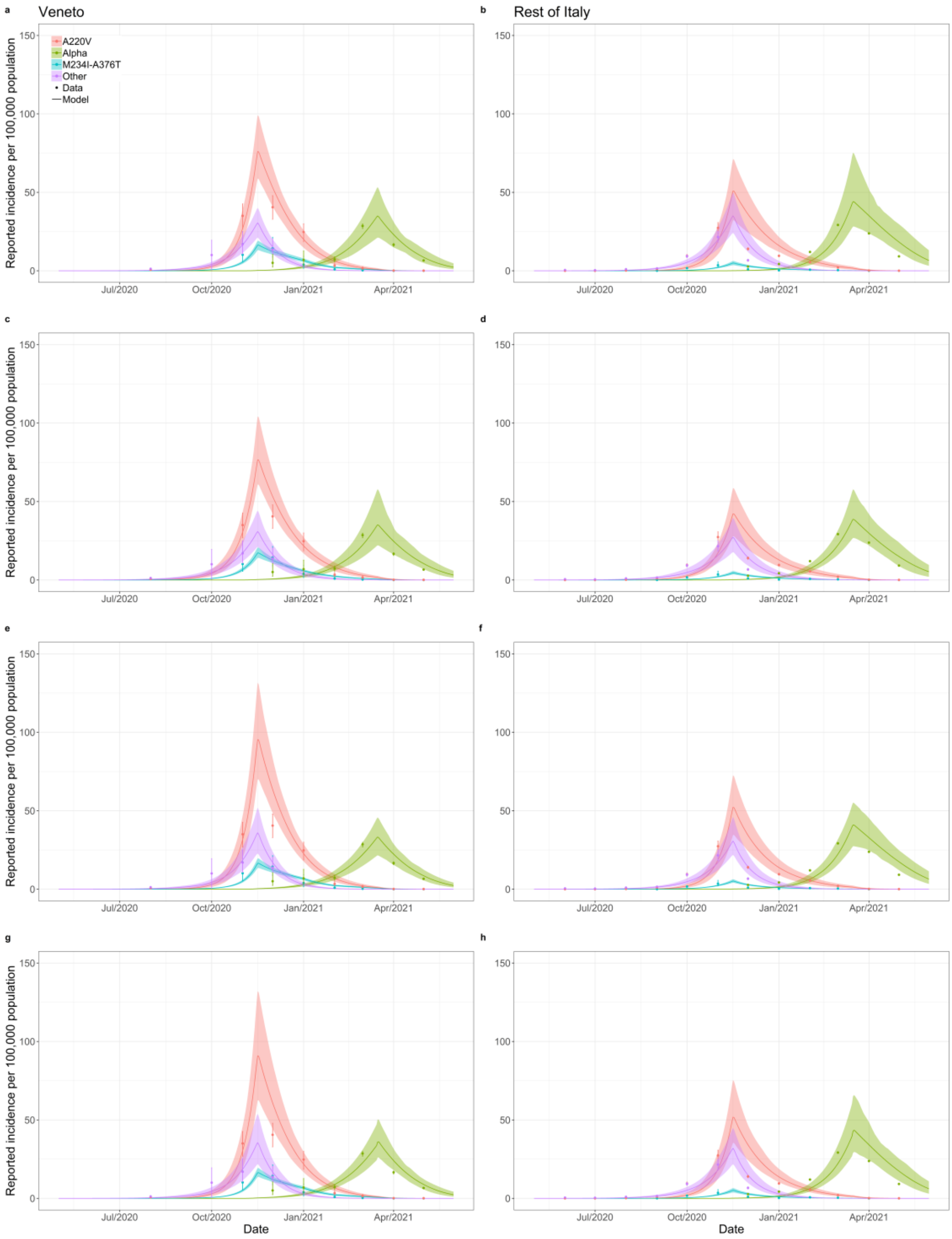

Supplementary Figure 6 | Transmission dynamics of SARS-CoV-2 variants (A220V, M234I-A376T, Alpha and other) in Veneto and the rest of Italy under different testing scenarios. **a)** Veneto and **b)** the rest of Italy estimated daily reported incidences per 100,000 population under the baseline testing scenario (Symptomatic cases test with a probability ρ and isolate if the test result is positive); **c)** Veneto and **d)** the rest of Italy estimated daily reported incidences per 100,000 population assuming that all symptomatic individuals isolate and that asymptomatic individuals test with a probability ρ and isolate if they receive a positive result; **e)** Veneto and **f)** the rest of Italy estimated daily reported incidences per 100,000 population assuming symptomatic individuals test and isolate if they receive a positive result, and that asymptomatic individuals test with a probability ρ and isolate if they receive a positive result; **g)** Veneto and **h)** the rest of Italy estimated daily reported incidences per 100,000 population assuming that asymptomatic and symptomatic individuals test with the same probability ρ and isolate if they receive a positive result. Model reported incidences are fitted from observed reported incidence data assuming a Negative Binomial likelihood (point and 95% binomial CI). Estimates are the mean and 95% CrI (solid line and shaded region), obtained from the 2.5% and 97.5% percentiles of 100 samples of the posterior distributions. ches

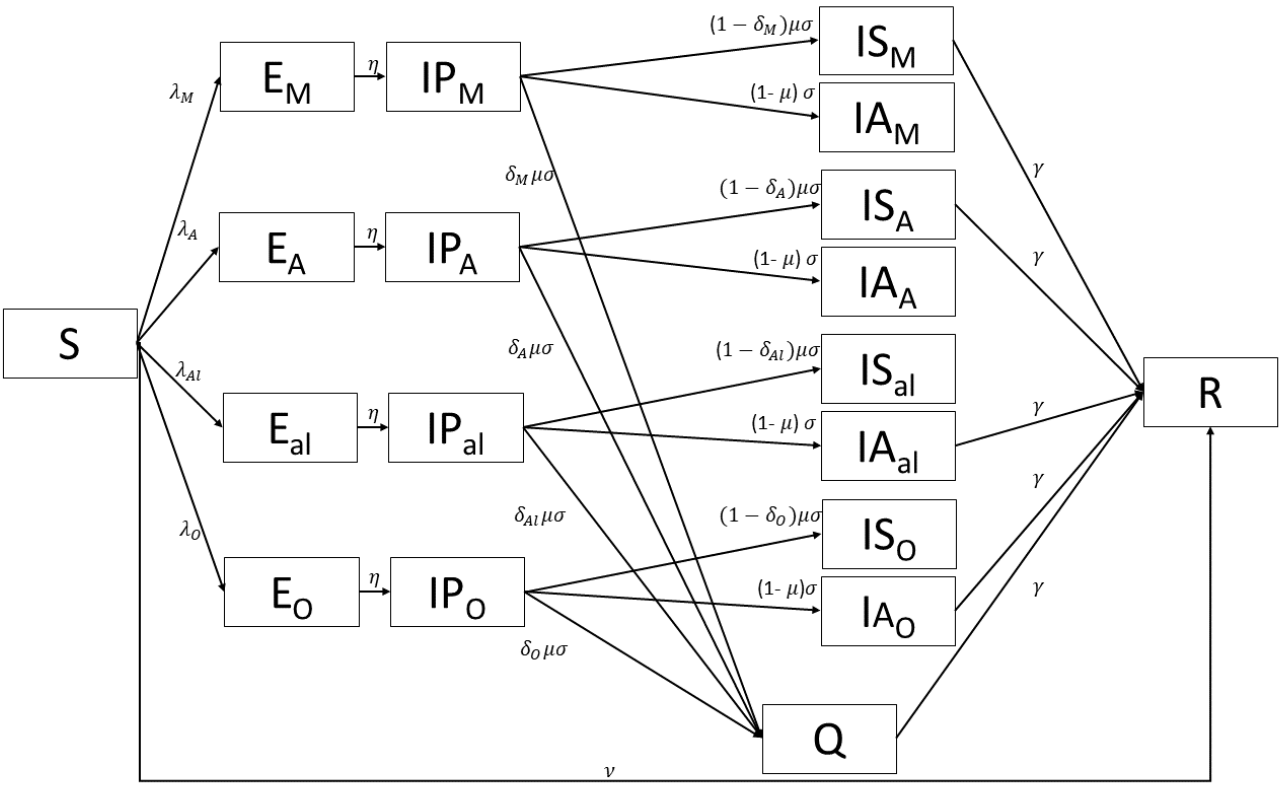

Supplementary Figure 7 | Flow diagram of the transmission model used to reproduce the dynamics of the discordant variant M234I-A376T and the concordant variants A220V, Alpha and other, in Veneto and the rest of Italy. Susceptible individuals are denoted S and are infected at rate $\lambda_Y = \frac{\beta_Y(IP_Y+IS_Y+IA_Y)}{S_0}$. Upon infection, individuals are latent with the virus (E compartment) for an average of $1/\eta$ days, after which individuals are viraemic but asymptomatic (IP_Y compartment) for an average of $1/\sigma$. From there, a proportion $(1-\mu)$ remain asymptomatic and infectious (IA_Y compartment) whilst the remaining proportion (μ) develop symptoms, of which $(1-\delta_Y)$ become infectious (IS_Y compartment) and δ_Y proportion are detected, reported and isolate (Q compartment). The infectious period lasts on average $1/\gamma$ days, and then individuals recover and test negative (R compartment). Susceptible individuals are vaccinated and enter the R compartment at rate ν. Subscript Y refers to the virus variant (M = M234I-A376T, A= A220V, Al = Alpha, O other variants).

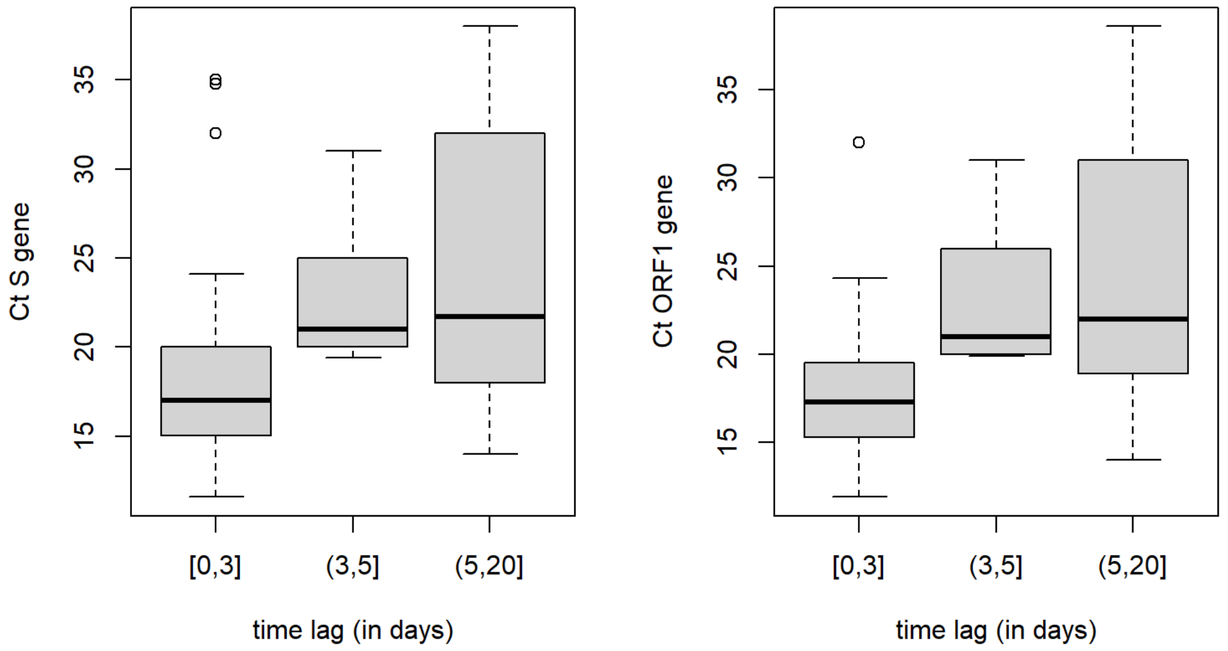

Supplementary Figure 8| Distribution of Ct values according to days from symptom onset: Boxplots of Ct values of S (left panel) and ORF1 (right panel) gene recorded at increasing time intervals (in days) from symptoms onset to day of testing 0-3 days, 4-5 days and above 5 days. P-value for Kruskall-Wallis test is 0.004 for Ct of ORF1 gene and 0.018 for Ct of S gene. n=61 biologically independent test samples. Box plots show the centre line as the median, box limits as the lower and upper quartiles, whiskers as 1.5 times the interquartile range or, if no points exceed this distance, as minimum and maximum values. Where whiskers are 1.5 times the interquartile range, points show data values exceeding this range

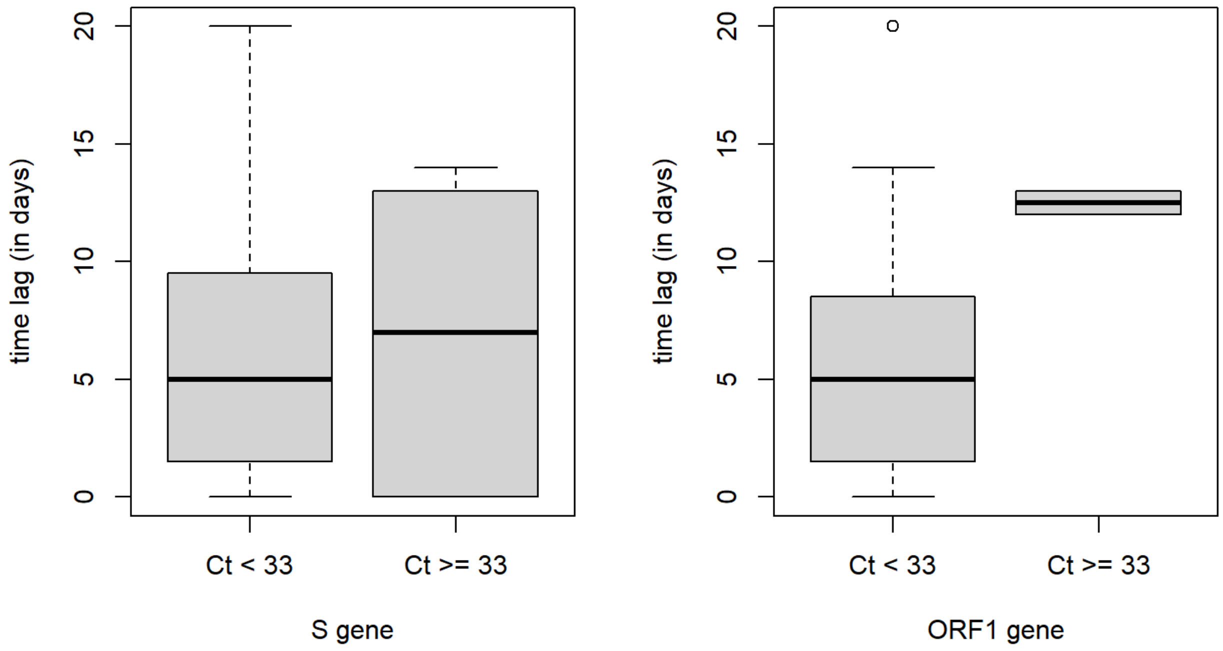

Supplementary Figure 9| Distribution of Ct values above and below 33 according to time intervals: Boxplots of time interval (in days) between time of testing and day of symptom onset against different levels of Ct values Ct < 33 and Ct >= 33 for S (left panel) and Ct ORF1 (right panel) gene. P-value for two-sided Wilcoxon-Mann-Whitney test is 1.0 for Ct S gene and 0.2 for ORF1 gene. n=61 biologically independent test samples. Box plots show the centre line as the median, box limits as the lower and upper quartiles, whiskers as 1.5 times the interquartile range or, if no points exceed this distance, as minimum and maximum values. Where whiskers are 1.5 times the interquartile range, points show data values exceeding this range

Supplementary Tables

Supplementary Table 1 | Estimates of antigen test sensitivity according to Ct values. Sensitivity estimates of antigen test at increasing Ct value for the S and ORF1 gene generated by a logistic regression model, with 95% confidence intervals. For each Ct threshold, the amount of concordant (Conc) and discordant (Disc) samples is reported.

Ct S gene						Ct ORF1 gene					
< value	sensitivity	Lower 95% confidence bound	upper 95% confidence bound	Conc	Disc	< value	sensitivity	Lower 95% confidence bound	upper 95% confidence bound	Conc	Disc
20	94.2	86.2	100.0	27	1	20	96.9	90.7	100.0	26	0
21	93.4	85.5	100.0	31	2	21	96.4	90.2	100.0	31	0
22	92.7	84.6	100.0	33	3	22	95.9	89.6	100.0	32	1
23	91.8	83.5	100.0	37	5	23	95.3	88.6	100.0	36	3
24	90.8	82.3	99.4	37	5	24	94.6	87.7	100.0	37	3
25	89.7	80.9	98.6	38	6	25	93.8	86.7	100.0	38	4
26	88.6	79.4	97.7	40	6	26	92.9	85.3	100.0	40	5
27	87.3	77.8	96.7	40	8	27	91.9	83.9	99.9	40	5
28	85.9	76.0	95.7	40	9	28	90.8	82.4	99.2	40	6
29	84.3	74.1	94.5	41	9	29	89.5	80.6	98.3	40	7
30	82.6	72.1	93.2	41	9	30	88.0	78.7	97.3	40	7
31	80.8	70.0	91.7	42	9	31	86.4	76.8	96.1	41	7
32	78.9	67.7	90.0	42	9	32	84.7	74.7	94.7	41	7
33	76.8	65.4	88.1	42	10	33	82.7	72.4	93.0	42	9
34	74.5	62.9	86.0	42	12	34	80.6	69.8	91.3	42	11
35	72.1	60.3	83.8	42	15	35	78.2	67.0	89.4	42	11
36	69.6	57.6	81.5	42	16	36	75.7	64.0	87.4	42	11
37	66.9	54.8	79.2	42	17	37	72.9	60.8	85.0	42	11
38	64.2	52.0	76.7	42	17	38	70.0	57.5	82.4	42	11

Supplementary Table 2 | Distribution of Ct values of samples. Welch Two Sample t-test of the distribution of Ct values of samples with concordant and discordant molecular / antigen test result. The hypothesis that the distributions of Ct values from molecular + / antigen + and discordant molecular + / antigen - test results are equal is rejected at the 5% significance level.

	Mean of Concordant	*Mean of Discordant*	*p-value*
Ct S gene all	*17.9*	*27.8*	*4.1e-06*
Ct S gene < 33	*17.9*	*24.4*	*9.0e-04*
Ct ORF1 gene all	*18.3*	*28.1*	*6.8e-05*
Ct ORF1 gene < 33	*18.3*	*26.3*	*1.5e-04*

Supplementary Table 3| Antigen sensitivity against variants with and without the amino-acid substitutions M234I and A376T, mapping to regions of the SARS-CoV-2 N protein containing epitopes that function as the target of capture antibodies in antigen tests.

	Variants M234I and A376T mutations (N = 3)	Variants without M234I and A376T mutations (N = 14)
Discordant variant (N = 8)	3	5
Concordant variant (N = 9)	0	9
Antigen test sensitivity (Mean and exact binomial 95% confidence interval)	0% (0-56.1%)	64.3% (38.8- 83.7%)

Supplementary Table 4 | Antigen sensitivity against variants with and without any mutation mapping to regions of the SARS-CoV-2 N protein containing epitopes that function as the target of capture antibodies in antigen tests.

	With mutations mapping to B cell epitopes (N = 9)	Without mutations mapping to B cell epitopes (N = 8)
Discordant variant (N = 8)	7	1
Concordant variant (N = 9)	2	7
Antigen test sensitivity (Mean and exact binomial 95% confidence interval)	22.2% (6.3-54.7%)	87.5 % (52.9-99.4%)

Supplementary Table 5 | Description of the concordant and discordant variants.

Name	Description
Variants without M234I and A376T mutations (Concordant)	
A220V	Genetic sequences obtained from GISAID containing the conservative A220V mutation.
Alpha	Genetic sequences obtained from GISAID from the B.1.1.7 pango lineage, against which antigen test sensitivity has been shown [2].
Other	All other genetic sequences obtained from GISAID.
Variants with M234I and A376T mutations (Discordant)	
M234I-A376T	Genetic sequences obtained from GISAID containing the M234I-A376T mutations.

Supplementary Table 6| Posterior mean and 95% CrI parameter estimates for model variants assuming different testing scenarios. Estimates are obtained from the 2.5% and 97.5% percentiles of the posterior distributions.

Parameter	Symptomatic cases test with probability ρ and isolate if the test result is positive.	All symptomatic cases test and isolate. Asymptomatic infections test with probability ρ and isolate if the test result is positive.	All symptomatic cases test and isolate if the test result is positive. Asymptomatic infections test with probability ρ and isolate if the test result is positive.	Symptomatic and asymptomatic infections test with probability ρ and isolate if the test result is positive.
R_{0M}	1.38 (1.3-1.43)	1.29 (1.25-1.34)	1.31 (1.25-1.37)	1.31 (1.26-1.38)
R_{0A}	1.45 (1.39-1.51)	1.36 (1.31-1.41)	1.43 (1.37-1.49)	1.43 (1.38-1.5)
R_{0O}	1.26 (1.23-1.31)	1.22 (1.19-1.26)	1.24 (1.2-1.29)	1.24 (1.2-1.28)
R_{0Al}	2.26 (2.16-2.37)	1.98 (1.9-2.07)	2.18 (2.08-2.29)	2.18 (2.08-2.3)
ρ	92.65% (72.84-99.82)	81.45% (45.72-99.23)	44.42% (6.42-87.05)	76.44% (58.31-93.94)
ω_1	36.56% (33.68-39.16)	34.75% (32.26-37.31)	39.04% (36.2-41.79)	39.03% (36.08-41.75)
ω_2	51.77% (47.29-56.01)	49.08% (45.03-53.32)	53.04% (49.1-57.06)	53.27% (49.26-57.3)
$I0_M$ $Italy$	105.41 (54.63-167.04)	114.41 (54.01-188.64)	154.49 (79.25-255.62)	152.95 (72.34-259.84)
$I0_A$ $Italy$	0.59 (0.06-2.42)	1.3 (0.14-5.26)	0.37 (0.03-1.56)	0.36 (0.03-1.63)
$I0_O$ $Italy$	88.27 (21.26-193.9)	74.05 (16.36-170.27)	77.07 (15.28-177.23)	78.44 (18.52-183.75)
$I0_{Al}$ $Italy$	88.34 (31.44-180.4)	122.6 (52.43-220.11)	90.49 (32.02-178.88)	91.32 (33.77-178.11)
$I0_M$ $Veneto$	24.68 (13.5-37.19)	21.54 (10.92-33.82)	21.04 (10.59-33.34)	21.24 (11.36-33.59)
$I0_A$ $Veneto$	0.07 (0.01-0.27)	0.2 (0.02-0.83)	0.05 (0-0.2)	0.05 (0-0.21)
$I0_O$ $Veneto$	6.29 (1.44-14.89)	6.95 (1.39-16.34)	6.3 (1.21-15.25)	6.35 (1.41-15.14)
$I0_{Al}$ $Veneto$	21.86 (11.31-34.84)	17.77 (8.31-29.8)	20.56 (9.84-34.18)	20.84 (10.16-34.6)
κ	0.05 (0.01-0.12)	0.04 (0.01-0.1)	0.05 (0.01-0.11)	0.05 (0.01-0.11)
Log-likelihood	-265.3 (-274.5- -258.6)	-261.1 (-270.0- -255.1)	-262.0 (-270.4- -255.8)	-262.5 (-271.2- -256.2)

Supplementary Table 7| Posterior mean and 95% CrI parameter estimates for model sensitivity analyses. ϕAg = Antigen test sensitivity; $1/\eta$ = latency period; $1/\sigma$ = pre-symptomatic infectious period; $1/\gamma$ = infectious period; ζ = vaccine efficacy against infection; α = reduction in transmission of symptomatic infectious individuals due to reduced exposure. Estimates are obtained from the 2.5% and 97.5% percentiles of the posterior distributions.

Model variant	1 (baseline)	2	3	4	5	6	7	8
$\boldsymbol{\phi_{Ag}}$	68.9%	64.3%	87.5%	68.9%	68.9%	68.9%	68.9%	68.9%
$\boldsymbol{\frac{1}{\eta}}$	1.31	1.31	1.31	1.31	2.15	1.31	1.31	1.31
$\boldsymbol{\frac{1}{\sigma}}$	3.79	3.79	3.79	4.29	2.95	3.79	3.79	3.79
$\boldsymbol{\frac{1}{\gamma}}$	2.1	2.1	2.1	1.6	2.1	2.1	2.1	2.1
$\boldsymbol{\zeta}$	100%	100%	100%	100%	100%	80%	70%	100%
$\boldsymbol{\alpha}$	-	-	-	-	-	-	-	9.28% (0.26-30.19)
$\boldsymbol{R_{0M}}$	1.38 (1.3-1.43)	1.38 (1.34-1.47)	1.44 (1.35-1.56)	1.45 (1.40-1.58)	1.37 (1.33-1.48)	1.38 (1.34-1.43)	1.38 (1.34-1.42)	1.35 (1.3-1.41)
$\boldsymbol{R_{0A}}$	1.45 (1.39-1.51)	1.45 (1.39-1.51)	1.48 (1.42-1.55)	1.48 (1.42-1.55)	1.47 (1.41-1.53)	1.45 (1.39-1.52)	1.45 (1.39-1.52)	1.4 (1.3-1.47)
$\boldsymbol{R_{0O}}$	1.26 (1.23-1.31)	1.26 (1.23-1.32)	1.29 (1.24-1.35)	1.29 (1.25-1.36)	1.27 (1.23-1.32)	1.26 (1.23-1.31)	1.26 (1.23-1.31)	1.22 (1.15-1.28)
$\boldsymbol{R_{0Al}}$	2.26 (2.16-2.37)	2.26 (2.16-2.44)	2.43 (2.22-2.74)	2.41 (2.28-2.77)	2.30 (2.19-2.51)	2.26 (2.16-2.38)	2.26 (2.16-2.37)	2.21 (2.1-2.34)
$\boldsymbol{\rho}$	92.65% (72.84-99.82)	92.07% (61.13-99.84)	66.36% (25.32-99.44)	87.41% (30.46-99.79)	91.13% (55.08-99.72)	93.39% (78.21-99.73)	93.24% (77.66-99.69)	89.32% (33.56-99.8)
$\boldsymbol{\omega_1}$	36.56% (33.68-39.16)	36.16% (31.6-39.12)	29.03% (10.3-39.2)	36.18% (14.44-40.63)	37.28% (30.34-40.4)	36.77% (33.81-39.43)	36.74% (33.82-39.39)	35.41% (17.93-39.06)
$\boldsymbol{\omega_2}$	51.77% (47.29-56.01)	51.16% (44.1-55.99)	40% (6.58-55.85)	51.54% (16.28-58.8)	52.13% (44.92-57.02)	52.69% (48.37-56.82)	53.04% (48.68-57.05)	50.25% (21.82-56.17)
$\boldsymbol{I0_M\ Italy}$	105.41 (54.63-167.04)	103.6 (53.69-168.3)	96.14 (47.23-161.63)	80.96 (41.39-131.89)	118.92 (60.74-190.55)	104.16 (53.64-166.51)	104.1 (55.22-166.63)	96.03 (47.8-157.14)

$I0_A$ $Italy$	0.59 (0.06-2.42)	0.68 (0.06-2.78)	0.91 (0.04-4.15)	0.89 (0.07-3.71)	0.52 (0.05-2.3)	0.55 (0.05-2.38)	0.58 (0.05-2.34)	0.69 (0.06-3.11)
$I0_O$ $Italy$	88.27 (21.26-193.9)	90.84 (21.28-202.59)	107.05 (21.49-250.25)	100.47 (21.19-223.63)	86.3 (19.73-196.86)	89.59 (20.96-199.96)	88.15 (18.22-200.93)	92.82 (22.15-210.64)
$I0_{Al}$ $Italy$	88.34 (31.44-180.4)	91.38 (33.42-183.45)	109.25 (32.36-238.61)	100.33 (35.36-210.49)	88.35 (32.67-184.75)	88.71 (30.83-182.24)	88.66 (32.96-173)	94.29 (34.64-197.98)
$I0_M$ $Veneto$	24.68 (13.5-37.19)	24.31 (13.22-37.75)	24.27 (13.17-37.61)	25.22 (13.42-39.15)	23.92 (12.9-36.66)	24.31 (13.08-37.87)	24.35 (13.51-37.69)	24.65 (13.1-37.93)
$I0_A$ $Veneto$	0.07 (0.01-0.27)	0.07 (0.01-0.29)	0.07 (0-0.25)	0.09 (0.01-0.34)	0.06 (0.01-0.23)	0.06 (0.01-0.27)	0.07 (0.01-0.27)	0.07 (0.01-0.27)
$I0_O$ $Veneto$	6.29 (1.44-14.89)	6.1 (1.32-13.99)	5.99 (1.43-14.59)	6.83 (1.45-15.63)	6.01 (1.32-14.45)	6.31 (1.37-14.79)	6.24 (1.19-14.88)	6.33 (1.48-14.66)
$I0_{Al}$ $Veneto$	21.86 (11.31-34.84)	21.82 (10.28-35.03)	18.42 (6.17-33.06)	21.44 (9.26-35.09)	21.53 (10.34-34.83)	21.88 (11.15-35.14)	21.94 (10.91-34.96)	21.56 (9.98-34.99)
κ	0.05 (0.01-0.12)	0.05 (0.01-0.11)	0.07 (0.02-0.14)	0.06 (0.01-0.13)	0.05 (0.01-0.11)	0.05 (0.01-0.12)	0.05 (0.01-0.12)	0.06 (0.01-0.12)

Supplementary Table 8| Antigen test sensitivity according to days intervals from symptom onset. Test sensitivity at increasing time interval from symptom onset with 95% confidence intervals. P-value = 0.22 for equality of proportions.

Days interval from symptom onset	Number of observations	Sensitivity (95% CI)
0-3 days:	29	75% (55 - 89)
4-5 days:	5	60% (17 - 93)
6-20 days:	18	50% (29 - 71)

References

1. Sempos, C.T. & Tian, L. Adjusting Coronavirus Prevalence Estimates for Laboratory Test Kit Error. *Am J Epidemiol* **190**, 109-115 (2021).

2. Rodgers, M.A. *et al.* Detection of SARS-CoV-2 variants by Abbott molecular, antigen, and serological tests. *J Clin Virol* **147**, 105080 (2022).
