# Supplementary material for: Impact of antigen test target failure and testing strategies on the transmission of SARS-CoV-2 variants
Source: Nat Commun. 2022 Oct 5;13:5870. doi: 10.1038/s41467-022-33460-0 (PMC9533294; doi:10.1038/s41467-022-33460-0)
Supplement: Supplementary file 3 — Description of Additional Supplementary Files [file 41467_2022_33460_MOESM3_ESM.pdf]

Description of Additional Supplementary Files

File Name: Supplementary Data 1
Description: ***N* gene mutation analysis. Monthly prevalence mutation)** Spatio-temporal analysis of the mutations affecting the *N* gene and identified in concordant and discordant samples. The monthly prevalence of each of the identified mutations is provided for Italy (excluded Veneto region) and for Veneto region within the 2020. ***N* protein)** Description of each sequenced concordant or discordant sample. GenBank and GISAID accession numbers of each sample is provided. Genomic and amino acid substitutions are reported for each of the sequenced sample, together with the relative Ct values, test results and symptoms onset data.

File Name: Supplementary Data 2
Description: **GISAID acknowledgment table.** List of all the sequences downloaded from GISAID and utilised in this study. For each sequence, GISAID accession ID is provided, together with the originating laboratories, the submitting laboratories and the authors.
