# Supplementary material for: Impact of antigen test target failure and testing strategies on the transmission of SARS-CoV-2 variants
Source: Nat Commun. 2022 Oct 5;13:5870. doi: 10.1038/s41467-022-33460-0 (PMC9533294; doi:10.1038/s41467-022-33460-0)
Supplement: Supplementary file 6 — Reporting Summary [file 41467_2022_33460_MOESM6_ESM.pdf]

nature portfolio

Corresponding author(s): Dr Ilaria Dorigatti

Last updated by author(s): Sep 4, 2022

Reporting Summary

Nature Portfolio wishes to improve the reproducibility of the work that we publish. This form provides structure for consistency and transparency in reporting. For further information on Nature Portfolio policies, see our Editorial Policies and the Editorial Policy Checklist.

Statistics

For all statistical analyses, confirm that the following items are present in the figure legend, table legend, main text, or Methods section.

n/a	Confirmed	
☐	☒	The exact sample size (*n*) for each experimental group/condition, given as a discrete number and unit of measurement
☐	☒	A statement on whether measurements were taken from distinct samples or whether the same sample was measured repeatedly
☐	☒	The statistical test(s) used AND whether they are one- or two-sided<br>*Only common tests should be described solely by name; describe more complex techniques in the Methods section.*
☐	☒	A description of all covariates tested
☐	☒	A description of any assumptions or corrections, such as tests of normality and adjustment for multiple comparisons
☐	☒	A full description of the statistical parameters including central tendency (e.g. means) or other basic estimates (e.g. regression coefficient) AND variation (e.g. standard deviation) or associated estimates of uncertainty (e.g. confidence intervals)
☐	☒	For null hypothesis testing, the test statistic (e.g. *F*, *t*, *r*) with confidence intervals, effect sizes, degrees of freedom and *P* value noted<br>*Give P values as exact values whenever suitable.*
☐	☒	For Bayesian analysis, information on the choice of priors and Markov chain Monte Carlo settings
☐	☒	For hierarchical and complex designs, identification of the appropriate level for tests and full reporting of outcomes
☐	☒	Estimates of effect sizes (e.g. Cohen's *d*, Pearson's *r*), indicating how they were calculated

Our web collection on statistics for biologists contains articles on many of the points above.

Software and code

Policy information about availability of computer code

Data collection	Data were collated using Microsoft Excel (v 16) from open source databases.
Data analysis	We used the following software for data analysis: Trimmomatic (v0.4044), BWA-MEM (v0.7.1745), Picard (v2.25.0), SAMtools (v1.1146), VarScan (v2.4.147), R (v 4.0.4) and RStudio (v 1.3.1073). Mathematical modelling was performed using R (v 4.1.1), RStudio (v 2021.09.0+351) and RStan (v 2.21.0). The code used to generate the modelling results is available on Github at https://github.com/bnc19/COV_Italy_multistrain.

For manuscripts utilizing custom algorithms or software that are central to the research but not yet described in published literature, software must be made available to editors and reviewers. We strongly encourage code deposition in a community repository (e.g. GitHub). See the Nature Portfolio guidelines for submitting code & software for further information.

Data

Policy information about availability of data

All manuscripts must include a data availability statement. This statement should provide the following information, where applicable:

- Accession codes, unique identifiers, or web links for publicly available datasets
- A description of any restrictions on data availability
- For clinical datasets or third party data, please ensure that the statement adheres to our policy

The SARS-CoV-2 genomic sequences of the concordant and of the discordant samples are available in the Supplementary Data 1, together with their relative

GenBank and GISAID accession numbers. The GISAID acknowledgements table is provided as the Supplementary Data 2, where all the GISAID accession numbers of the sequences downloaded from GISAID for this study are available. Data used in the modelling study are available on Github at https://github.com/bnc19/COV_Italy_multistrain. The original datasets are publicly available from https://github.com/italia/covid19-opendata-vaccini (data on the daily number of vaccine doses administered in each region of Italy) and from https://github.com/pcm-dpc/COVID-19 (data on the daily number of reported SARS-CoV-2 cases in each region of Italy and data on daily the number of antigen and molecular tests administered in each region of Italy)

Human research participants

Policy information about studies involving human research participants and Sex and Gender in Research.

Reporting on sex and gender	Information on the sex of participants was collected.
Population characteristics	3,290 patients aged 1-90+ years examined from the 15th of September to the 16th of October 2020 in the A&E and Infectious disease wards of the University Hospital of Padua (Italy) were initially included in the study. Only subjects aged 20 year and older who received both an antigen and a molecular test were included in the main analysis (1,387 subjects). Of these, 53% were female.
Recruitment	All the patients admitted to the A&E and Infectious disease wards of the University Hospital of Padua (Italy) from 15th September 2020 to 16th October 2020 (time interval of the study) who were subjected to both molecular and antigenic swabs. As this was a retrospective study and data was extract from the electronic medical record administrative database of the University Hospital of Padova there was no self-selection bias in the recruitment of participants. Individuals aged 0-19 years were predominantly tested with a molecular swab assay only and the few that were tested with both antigen and molecular swab assays were omitted from the analysis to avoid sampling biases.
Ethics oversight	The data were extracted from the electronic medical record administrative database (Galileo platform) of the University Hospital of Padova. The purpose and design of the study has been approved by the Local Ethics Committee of the Province of Padova (Italy) (Protocol Numbers: 69295, 0001609). The need for written informed consent was waived for patients because this is a retrospective study and this is in accordance with the Italian Drug Agency note 20 - March 2008 (GU Serie Generale no. 76 31/3/2008).

Note that full information on the approval of the study protocol must also be provided in the manuscript.

Field-specific reporting

Please select the one below that is the best fit for your research. If you are not sure, read the appropriate sections before making your selection.

☒ Life sciences ☐ Behavioural & social sciences ☐ Ecological, evolutionary & environmental sciences

For a reference copy of the document with all sections, see nature.com/documents/nr-reporting-summary-flat.pdf

Life sciences study design

All studies must disclose on these points even when the disclosure is negative.

Sample size	This is a retrospective diagnostic hospital-based validation study without assumptions on the sample size. All the patients admitted to the A&E and Infectious disease wards of the University Hospital of Padua (Italy) from 15th September 2020 to 16th October 2020 (the time interval of the study) were subjected to both molecular and antigenic swabs. Swab samples were collected both for patients and subjects who required SARS-CoV-2 testing for one of the following reasons: a) presence of symptoms indicating a possible SARS-CoV-2 infection (fever and/or cough and/or headache, diarrhoea, asthenia, muscle pain, joint pain, loss of taste or smell, or shortness of breath, with or without pneumonia); b) patients who were asymptomatic but had a contact with a confirmed SARS-CoV-2 case during the previous ten days. All subjects were tested within one hour from presentation with both an antigen and a molecular swab assay. For each individual information about their age, sex, date of sampling, symptoms, time of symptom onset, and Ct value of the molecular test were recorded.
Data exclusions	Individuals from 0-19 years old were predominantly tested with a molecular swab assay only and the few that were tested with both antigen and molecular swab assays were omitted from the analysis to avoid sampling biases.
Replication	This is a retrospective diagnostic validation study and replication is not applicable.
Randomization	Randomization is not relevant to our study because this is a retrospective diagnostic validation study
Blinding	Blinding is not relevant to our study because this is a retrospective diagnostic validation study

Reporting for specific materials, systems and methods

We require information from authors about some types of materials, experimental systems and methods used in many studies. Here, indicate whether each material, system or method listed is relevant to your study. If you are not sure if a list item applies to your research, read the appropriate section before selecting a response.

Materials & experimental systems

n/a	Involved in the study
☒	☐ Antibodies
☐	☒ Eukaryotic cell lines
☒	☐ Palaeontology and archaeology
☒	☐ Animals and other organisms
☒	☐ Clinical data
☒	☐ Dual use research of concern

Methods

n/a	Involved in the study
☒	☐ ChIP-seq
☒	☐ Flow cytometry
☒	☐ MRI-based neuroimaging

Eukaryotic cell lines

Policy information about cell lines and Sex and Gender in Research

Cell line source(s)	Cell line was obtained from the ATCC (ATCC-CCL-81 Kidney; African Green Monkey (Cercopithecus aethiops))
Authentication	NOT APPLICABLE
Mycoplasma contamination	The cell line tested negative for mycoplasma contamination
Commonly misidentified lines (See ICLAC register)	NOT APPLICABLE
